# Multi-Omics and Experimental Insights into the Protective Effects of Sesquiterpenoid Lactones from *Eupatorium lindleyanum* DC. in Acute Lung Injury: Regulation of PI3K-Akt and MAPK-NF-κB Pathways

**DOI:** 10.3390/ph18101523

**Published:** 2025-10-10

**Authors:** Chen Luo, Yan Yang, Lian Xia, Keyun Zhou, Chuanxin Liu, Ling Yao, Weiguo Cao, Xianqin Luo

**Affiliations:** 1 College of Traditional Chinese Medicine, Chongqing Medical University, Chongqing 400016, China; 2 Chongqing Key Laboratory of Traditional Chinese Medicine for Prevention and Cure of Metabolic Diseases, Chongqing 400016, China; 3 College of Traditional Chinese Medicine, Chongqing University of Chinese Medicine, Chongqing 402760, China; yaoling@cqctcm.edu.cn; 4 College of Chinese Materia Medica, Chongqing University of Chinese Medicine, Chongqing 402760, China

**Keywords:** *Eupatorium lindleyanum* DC., sesquiterpene lactones, acute lung injury, multi-omics integration, gut-lung axis, network pharmacology

## Abstract

**Background:** Acute lung injury (ALI) is a life-threatening respiratory condition and one of the leading causes of mortality worldwide, accounting for approximately 20% of global annual deaths. Despite its high prevalence and severity, effective therapeutic options remain limited. *Eupatorium lindleyanum* DC., a traditional medicinal herb, has demonstrated therapeutic potential against pulmonary diseases, particularly ALI, in both clinical and experimental settings. However, the protective effects and underlying mechanisms of its characteristic sesquiterpene lactone components against ALI remain unclear. **Objective:** This study aimed to evaluate the protective effects of sesquiterpene lactones from *Eupatorium lindleyanum* DC. (SLEL) against lipopolysaccharide (LPS)-induced ALI both in vivo and in vitro. Furthermore, it sought to elucidate the underlying mechanisms by integrating network pharmacology, multi-omics approaches (transcriptomics, metabolomics, and 16S rRNA sequencing), and various molecular biology techniques. **Results:** SLEL significantly attenuated inflammatory injury in alveolar epithelial cells and alleviated pulmonary edema, hemorrhage, and inflammatory infiltration in rats, accompanied by reduced TNF-α, IL-6, and IL-1β levels and improved lung injury indices. Mechanistically, SLEL exerted dual suppression of the PI3K-Akt and MAPK-NF-κB pathways. Network pharmacology, molecular docking, and UPLC-MS analyses identified Eupalinolide A and Eupalinolide K as potential bioactive constituents, which were further validated to inhibit phosphorylation of key signaling proteins, thereby partially accounting for SLEL’s pharmacological effects. Multi-omics integration further revealed that SLEL restored bile acid metabolism, reshaped gut microbial diversity, and reconstructed the microbiota–metabolite–inflammatory cytokine network, thereby maintaining gut–lung axis homeostasis and enhancing anti-inflammatory effects. **Conclusions:** SLEL alleviates ALI through multi-component synergistic actions that suppress pro-inflammatory signaling and modulate the gut–lung axis. These findings highlight the potential of SLEL as a promising therapeutic candidate for the treatment of ALI.

## 1. Introduction

Acute lung injury (ALI) is defined as a severe respiratory syndrome characterized clinically by acute hypoxemic respiratory failure [1]. Notably, ALI represents a critical stage in a pathological continuum that, when left untreated or inadequately managed, often progresses to acute respiratory distress syndrome (ARDS) [2]. From an epidemiological perspective, ALI remains a major contributor to global mortality, accounting for approximately 20% of annual deaths worldwide, as reported by the Global Burden of Disease Study [3]. Current therapeutic strategies for ALI include optimized mechanical ventilation [4], extracorporeal membrane oxygenation (ECMO) [5], pharmacological interventions with corticosteroids and nonsteroidal anti-inflammatory drugs (NSAIDs) [6], advanced gene-editing techniques [7], and emerging neuroimmune modulators [8]. However, these approaches have not significantly reduced the mortality associated with ALI and ARDS. Furthermore, they pose substantial challenges to equitable healthcare access, especially in low-income populations, and are often associated with adverse effects such as tissue trauma and systemic toxicity [9]. Therefore, there is an urgent clinical need to develop natural therapeutic agents with effective protective potential against ALI.

*Eupatorium lindleyanum* DC. (EL), a sesquiterpene lactone-rich medicinal plant has garnered attention for its historical and pharmacological relevance, particularly during the 2003 SARS and 2020 COVID-19 outbreaks [10]. Sesquiterpene lactones, widely studied in pharmacologically active plants, have demonstrated multi-target therapeutic effects including anti-inflammatory, antioxidant, immunomodulatory, neuroprotective, organ-protective, and antitumor activities [11,12]. Existing studies have shown that ethanol extracts of EL possess anti-inflammatory properties [13] and confer protection against ALI through modulation of the TLR4/NF-κB/NLRP3 pathway and remodeling of gut microbiota [14]. However, the specific protective effects and underlying mechanisms of its characteristic components—sesquiterpene lactones in the context of ALI remain poorly understood.

Based on this evidence, we hypothesize that SLEL can attenuate LPS-induced ALI by inhibiting inflammation-related signaling pathways and reducing the release of pro-inflammatory cytokines. To test this hypothesis, we systematically investigated the protective effects of SLEL using LPS-induced ALI models in both A549 cells and rats, and applied integrated multi-omics analyses to identify its molecular targets.

## 2. Results

### 2.1. Chemical Composition in SLEL Through UPLC-Q/TOF/MSn

Analysis of SLEL using UPLC-Q/TOF-MSn in positive/negative ion modes identified nine sesquiterpene lactones as major constituents (Supplementary Figure S1). Molecular formulas were determined based on adduct ions: [M+H]^+^ or [M+Na]^+^ (positive mode) and [M−H]^−^ or [M+HCOO]^−^ (negative mode) (Supplementary Table S2). Notably, a novel sesquiterpene lactone, tentatively named Eupalinolide P, was discovered, displaying structural homology to Eupalinolide A/B.

### 2.2. SLEL Attenuates LPS-Induced Inflammatory Cascade in Macrophages via Cytokine Suppression

**Figure 1:**
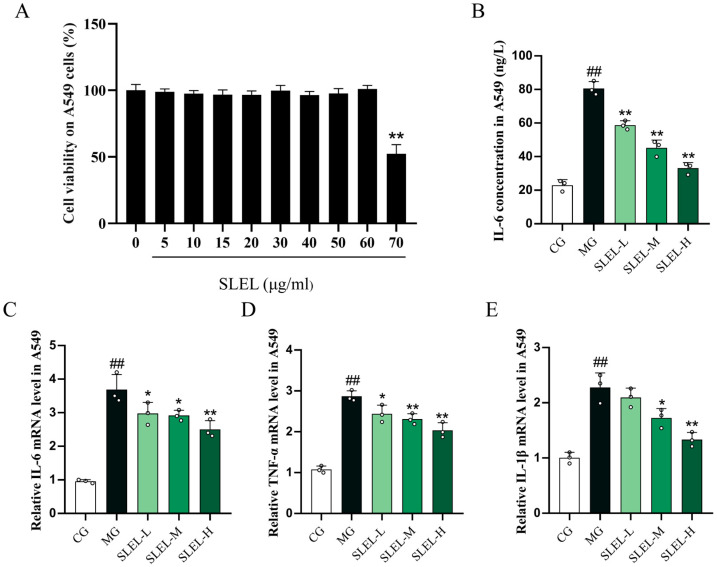
Effects of SLEL on cell viability and inflammatory responses in LPS-stimulated A549 cells. (**A**) CCK-8 assay showing the effect of SLEL (0–70 μg/mL) on A549 cells’ viability. (**B**) ELISA analysis of IL-6 secretion in A549 cells. (**C**) qPCR analysis of IL-6 mRNA levels in A549 cells. (**D**) qPCR analysis of TNF-α mRNA levels in A549 cells. (**E**) qPCR analysis of IL-1β mRNA levels in A549 cells. ^##^ *p* < 0.01 compared to the CG. * *p* < 0.05, ** *p* < 0.01 compared to the MG.

CCK-8 assays confirmed SLEL’s non-cytotoxic profile in A549 cells (5–60 μg/mL), guiding anti-inflammatory dose selection (Figure 1A). In LPS-stimulated A549 cells, SLEL (15, 30, and 60 μg/mL) significantly suppressed IL-6 secretion (*p* < 0.01, Figure 1B) and downregulated the transcriptional levels of IL-6, IL-1β, and TNF-α (*p* < 0.01, Figure 1C–E).

### 2.3. SLEL Decreased the Inflammation in ALI Rats

**Figure 2:**
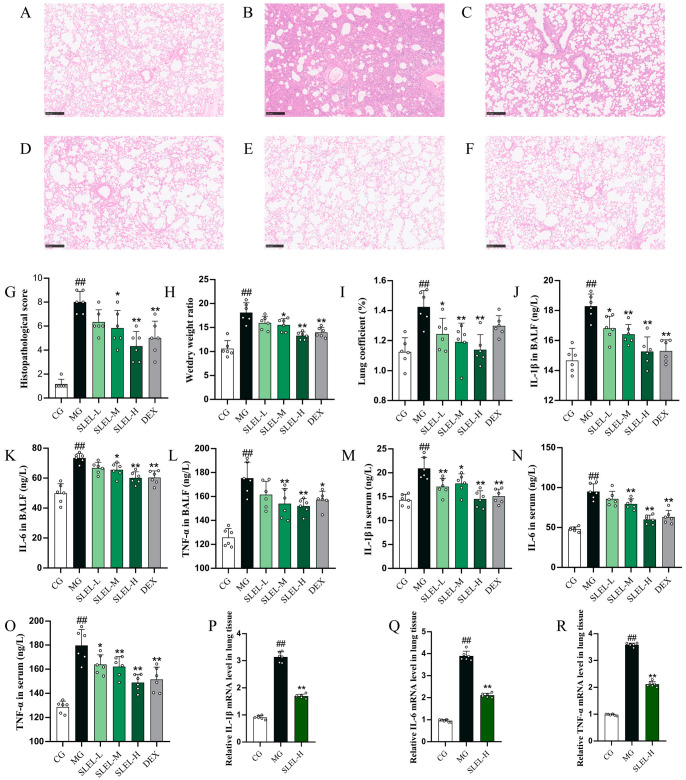
Effect of SLEL on LPS-induced lung pathological injury and inflammatory cytokines in ALI rats. Representative photomicrographs of HE staining of lung tissues in each group (magnification, ×100): CG group (**A**), MG group (**B**), SLEL-L group (**C**), SLEL-M group (**D**), SLEL-H group (**E**), DEX group (**F**). (**G**) Lung injury scoring scale (n = 6). (**H**) Lung wet/dry weight ratio (n = 6). (**I**) Lung coefficient. The modulatory effects of SLEL on cytokine levels of IL-1β (**J**), IL-6 (**K**), and TNF-α (**L**) in BALF (n = 6), as well as cytokine levels of IL-1β (**M**), IL-6 (**N**), and TNF-α (**O**) in serum (n = 6), were examined. IL-1β (**P**), IL-6 (**Q**), and TNF-α (**R**) mRNA levels by real-time PCR in LPS-stimulated lung tissue with SLEL-H treatment (n = 6). ^##^ *p* < 0.01 compared to the CG. * *p* < 0.05, ** *p* < 0.01 compared to the MG.

H&E staining demonstrated that SLEL treatment markedly alleviated LPS-triggered pulmonary edema, hemorrhage, inflammatory infiltration, and alveolar wall thickening, with reduced lung injury scores (*p* < 0.01, Figure 2A–G). Consistent with this, SLEL dose-dependently lowered lung coefficient and wet/dry weight ratio (*p* < 0.01, Figure 2H,I). SLEL further suppressed pro-inflammatory cytokines (IL-6, IL-1β, TNF-α) in both BALF and serum (*p* < 0.01, Figure 2J–O), while downregulating their mRNA expression in lung tissue (*p* < 0.01, Figure 2P–R). These findings confirm SLEL’s therapeutic potential in mitigating LPS-induced pulmonary inflammation and structural damage.

### 2.4. Network Pharmacology and Molecular Docking Elucidate SLEL’s Anti-ALI Mechanisms

**Figure 3:**
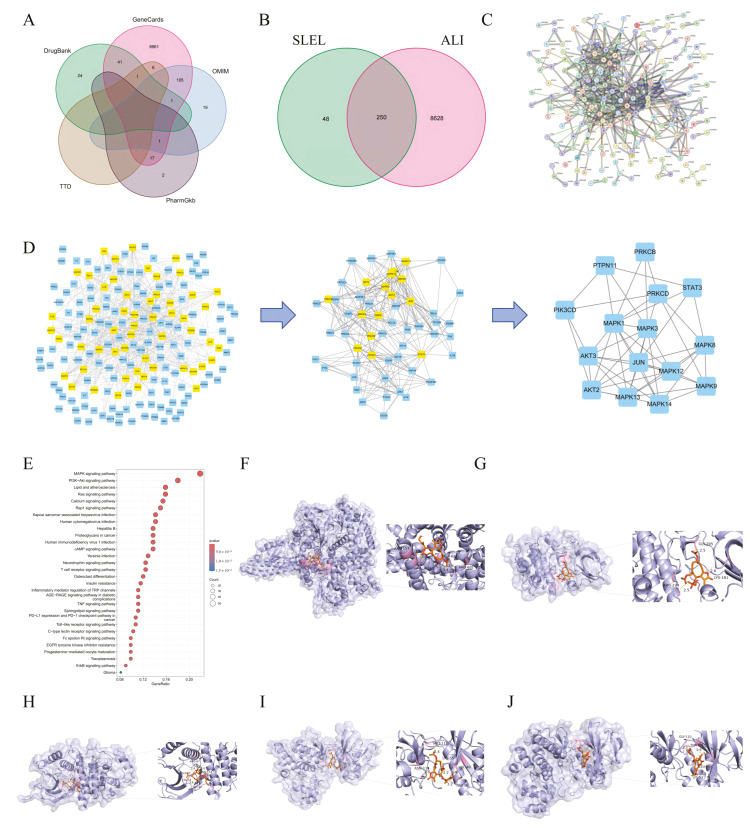
The potential drug target pathways and molecular docking results related to the ALI activity of SLEL. (**A**) ALI-related targets included in various platforms. (**B**) Overlap of SLEL potential targets and ALI-related targets. (**C**) The PPI network is constructed based on the overlapping gene targets of SLEL against ALI. (**D**) Identify the most critical PPI network by employing a scoring system. (**E**) Top 30 enriched KEGG pathways. (**F**) The optimal binding mode of Eupalinolide K bonded with PI3K. (**G**) The optimal binding mode of Eupalinolide A bonded with Akt. (**H**) The optimal binding mode of Eupalinolide A bonded with ERK. (**I**) The optimal binding mode of Eupalinolide A bonded with JNK. (**J**) The optimal binding mode of Eupalinolide A bonded with P38.

Network pharmacology analysis identified 250 overlapping targets between SLEL-derived compounds (298) and ALI-related genes (8878) (Figure 3A,B). A Protein–Protein Interaction (PPI) network (27 nodes, 47 edges) highlighted STAT3, MAPK family members (MAPK3/14/1/12/8/13/9), AKT2/3, and PI3K/JUN signaling nodes as core hub genes (Figure 3C,D). KEGG enrichment prioritized PI3K-Akt and MAPK pathways as key SLEL-ALI therapeutic axes (Figure 3E). Molecular docking further confirmed the strong binding affinity of SLEL compounds with key targets (binding energies ranging from −8.3 to −6.3 kcal/mol), particularly concentrated in the PI3K-Akt and MAPK signaling cascades, demonstrating their structural compatibility. Specifically, Eupalinolide K exhibited the optimal binding mode with PI3K (Figure 3F), whereas Eupalinolide A showed stable interactions with Akt, ERK, JNK, and P38 (Figure 3G–J), with corresponding binding energies of −6.3, −6.4, −7.7, −7.7, and −8.3 kcal/mol, respectively. These results mechanistically link the anti-inflammatory effects of SLEL to the PI3K-Akt and MAPK signaling pathways.

### 2.5. Transcriptomics Unravels SLEL’s Anti-ALI Pathway Modulation

**Figure 4:**
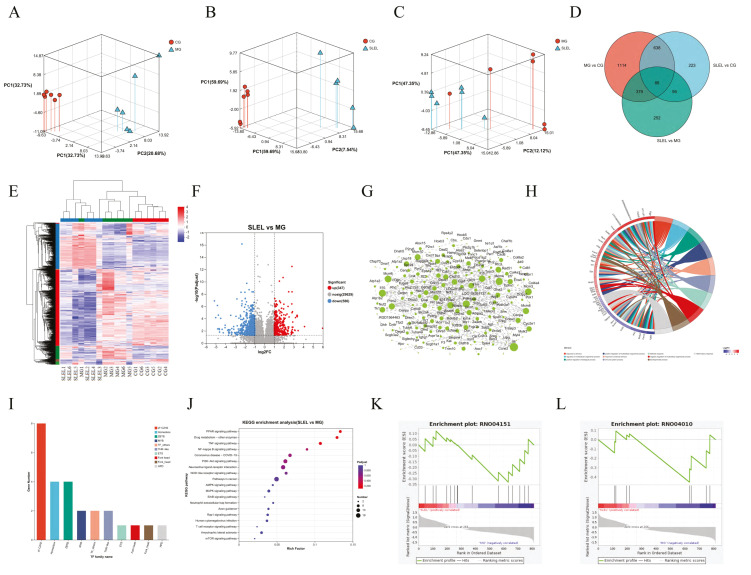
Analysis of target and pathway of SLEL against ALI based on transcriptomics. (**A**) Principal component analysis (PCA) of CG and MG groups. (**B**) PCA of CG and SLEL groups. (**C**) PCA of MG and SLEL groups. (**D**) Demonstrates a Venn diagram illustrating the number of differentially expressed genes (DEGs) among the three groups (MG Vs. CG, SLEL Vs. CG, and SLEL Vs. MG as well as the overlapping relationships among the comparative groups. (**E**) Depicts a clustering diagram of DEGs, with red denoting up-regulation, and blue indicating down-regulation. (**F**) Presents DEGs of SLEL Vs. MG, where red, blue, and gray represent up-regulated, down-regulated, and non-DEGs, respectively. (**G**) Constructs a PPI network based on the DEGs identified in the comparison between SLEL and MG. (**H**) Presents the GO enrichment chord diagram based on the DEGs identified in the comparison between SLEL and MG. (**I**) A family-based statistical analysis was conducted on the transcription factors that predict the relationship between SLEL and MG. (**J**) Exhibits KEGG enrichment results of SLEL Vs. MG DEGs. (**K**) Showcase the GSEA analysis of PI3K-Akt signaling pathway. (**L**) Showcase the GSEA analysis of MAPK signaling pathway.

**Figure 5:**
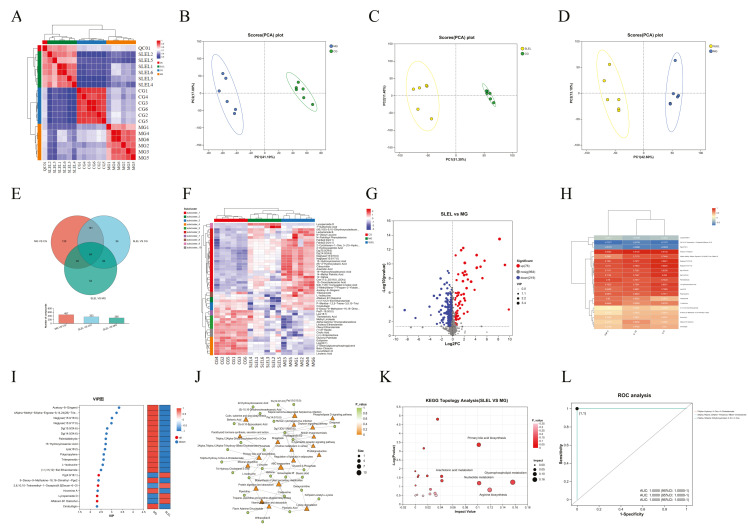
Analysis of target and pathway of SLEL against ALI based on metabolomics. (**A**) Shows a correlation heatmap of samples among CG, MG, and SLEL, with red indicating positive correlation and blue indicating negative correlation. Darker colors represent stronger correlations. (**B**) PCA of CG and MG groups. (**C**) PCA of CG and SLEL groups. (**D**) PCA of MG and SLEL groups. (**E**) Exhibits Venn diagrams illustrating differential metabolites among CG, MG, and SLEL. (**F**) Depicts a clustering diagram of differential metabolites, with red denoting up-regulation, and blue indicating down-regulation. (**G**) Depicts differential metabolites between SLEL and MG, with red, blue, and gray colors representing up-regulated, down-regulated, and non-differential metabolites, respectively. (**H**) Correlation analysis between differential metabolites and inflammatory cytokines. (**I**) Exhibits the 20 metabolites with the highest VIP values, showing the greatest differential expression between SLEL and MG. (**J**) Presents the KEGG enrichment analysis network diagram of differentially expressed metabolites between SLEL and MG. (**K**) Presents the KEGG topological analysis of differentially expressed metabolites between SLEL and MG. (**L**) Shows ROC results of metabolites.

Transcriptomic profiling of ALI rats revealed distinct transcriptional clustering among CG, MG, and SLEL-treated groups (Figure 4A–C). SLEL treatment induced 933 differentially expressed genes (DEGs), including 347 upregulated and 586 downregulated in lung tissue (Figure 4D–F), with PPI networks and functional analysis linking these DEGs to suppressed inflammatory markers (e.g., IL-17C/F, TNF, IL-10) (Figure 4G–H). Transcription factor families zf-C2H2, Homeobox, and ZBTB were prioritized as regulatory hubs (Figure 5I). Integrated KEGG and Gene set enrichment analysis (GSEA) confirmed concordant downregulation of PI3K-Akt and MAPK signaling—previously identified in network pharmacology—as central to SLEL’s therapeutic efficacy (Figure 4J–L), reinforcing dual-pathway targeting as the mechanistic cornerstone of SLEL against ALI.

### 2.6. Metabolomics Decodes SLEL’s Bile Acid-Mediated Anti-ALI Mechanism

Non-targeted fecal metabolomics in ALI rats revealed distinct metabolic separation among CG, MG, and SLEL-treated groups (Figure 5A–D). SLEL restored 295 dysregulated metabolites toward CG-like patterns (Variable importance in projection [VIP] > 1, *p* < 0.05). Correlation analysis with inflammatory cytokines TNF-α, IL-1β, and IL-6 further confirmed the close association between these metabolic changes and inflammatory responses. Notably, the downregulation of inflammation-related metabolites was particularly significant (Figure 5E–I). KEGG analysis highlighted primary bile acid biosynthesis as the most suppressed pathway, with pro-inflammatory bile acid derivatives uniformly reduced (Figure 5J,K). Receiver operating characteristic (ROC) validation of these metabolites showed perfect discriminative power (AUC = 1, Figure 5L), supporting SLEL’s dual action in attenuating intestinal flora-driven inflammation and immune dysregulation Via bile acid modulation.

### 2.7. 16S rRNA Analysis of SLEL’s Impact on Gut Microbiota in ALI

**Figure 6:**
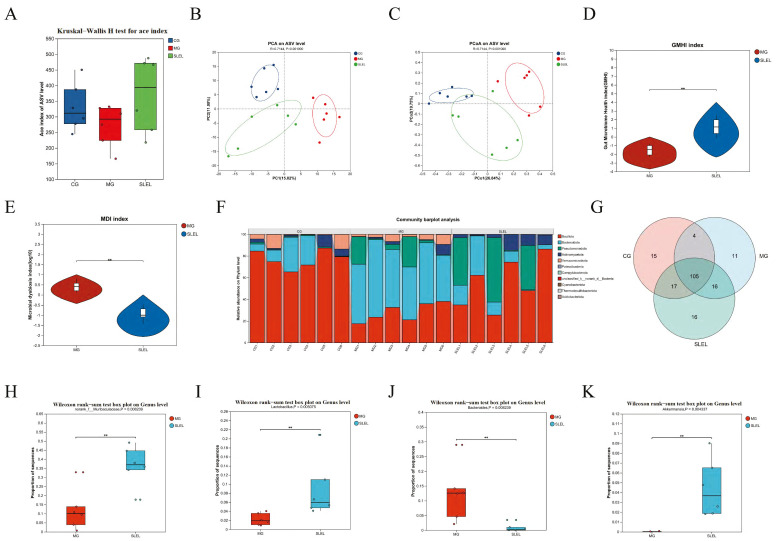
The Impact of SLEL on Gut Microbiota in the Context of ALI. (**A**) Presents Alpha Diversity Index Analysis for CG, MG, and SLEL. (**B**) PCA for CG, MG, and SLEL groups. (**C**) Principal Coordinate Analysis (PCoA) for CG, MG, and SLEL groups. (**D**) Exhibits the gut microbiota health index (GMHI) between SLEL and MG. (**E**) Exhibits the gut microbial dysbiosis index (MDI) between SLEL and MG. (**F**) Illustrates the differences in the phylum-level composition of the intestinal microbiota among CG, MG, and SLEL groups. (**G**) Exhibits Venn diagram illustrating the differences in genus-level intestinal microbiota composition among CG, MG, and SLEL groups. (**H**) Alterations in the relative abundance of *norank_f__Muribaculaceae*. (**I**) Alterations in the relative abundance of *Lactobacillus*. (**J**) Alterations in the relative abundance of *Bacteroides*. (**K**) Alterations in the relative abundance of *Akkermansia*. ** *p* < 0.01 compared to the MG.

To investigate SLEL’s anti-inflammatory mechanism linked to gut microbiota modulation, 16S rRNA sequencing revealed that SLEL administration enhanced microbial α-diversity (Figure 6A) and induced structural segregation in β-diversity (Figure 6B,C), with concurrent amelioration of dysbiosis (Figure 6D,E). Phylum-level profiles showed significant enrichment of *Bacillota*, *Pseudomonadota*, and *Actinomycetota*, alongside reduced *Bacteroidota* versus the MG (Figure 6F). Notably, genus-level taxa demonstrated SLEL-driven increases in *norank_f__Muribaculaceae*, *Lactobacillus*, and *Akkermansia*, while markedly suppressing *Bacteroides* (Figure 6G–K).

### 2.8. Integrated Multi-Omics Analysis Reveals SLEL’s Anti-ALI Mechanisms

**Figure 7:**
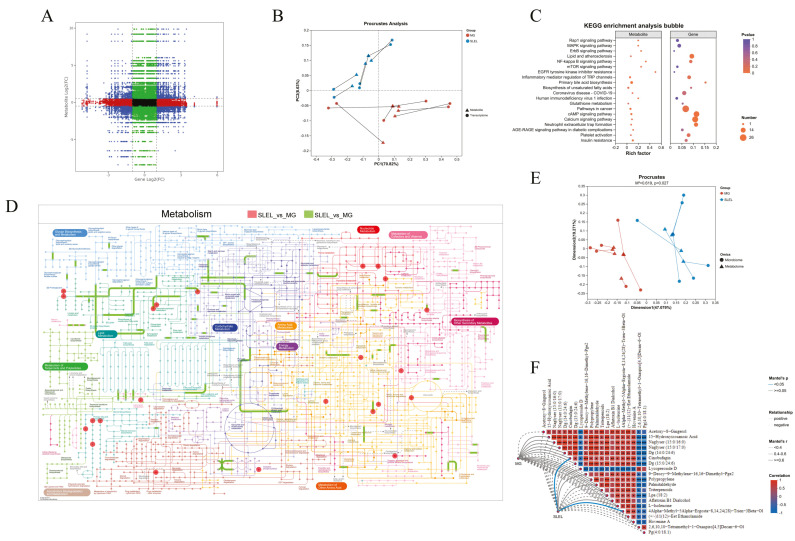

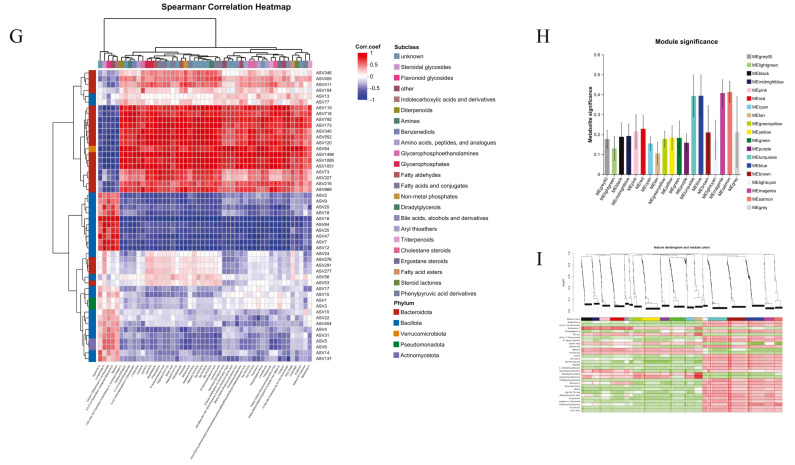
Integrated transcriptome and metabolome analysis, as well as microbiome and metabolome analysis. (**A**) Presents a correlation quadrant diagram. (**B**) Procrustes analysis of metabolome and transcriptome data between the SLEL and MG groups. (**C**) Exhibits KEGG enrichment results of SLEL vs. MG DEGs and differentially expressed metabolites. (**D**) Exhibits metabolism results of SLEL vs. MG groups. (**E**) Procrustes analysis of metabolome and microbiome data between the SLEL and MG groups. (**F**) Presents Mantel-test network heatmap of SLEL vs. MG groups. (**G**) Exhibits spearmanr correlation heatmap of metabolome and microbiome data between the SLEL and MG groups. (**H**) Presents module significance between SLEL and MG groups. (**I**) Further feature dendrogram and module color assignments analysis.

Transcriptomic-metabolomic integration identified strongly correlated differential mRNAs and metabolites (|r| > 0.8, p < 0.05, Figure 7A). Procrustes analysis confirmed structural alignment between metabolomic and transcriptomic profiles in SLEL versus MG (Figure 7B). KEGG enrichment highlighted NF-κB signaling as a key inflammation-related pathway (Figure 7C), while iPath mapping implicated primary bile acid biosynthesis in SLEL’s efficacy (Figure 7D). Further Procrustes analysis of microbiome-metabolome interactions revealed SLEL-driven structural shifts (Figure 7E). Mantel test-based network heatmaps showed that, compared with the MG group, SLEL restored the interaction network between gut microbiota and metabolites, which may represent an important mechanism underlying its anti-inflammatory effects. (Figure 7F). The correlation heatmap indicated that SLEL treatment reshaped the interactions between gut microbiota and metabolites, where Bacteroidota showed strong positive correlations with anti-inflammatory steroidal and triterpenoid metabolites, whereas Bacillota exhibited negative correlations with inflammation-related fatty acid and amino acid derivatives, suggesting that microbiota–metabolite interactions may contribute to its anti-inflammatory effects (Figure 7G). Weighted gene co-expression network analysis (WGCNA) identified that the pink, red, and magenta modules were significantly associated with the treatment group, suggesting their potential involvement in the anti-inflammatory effects. The metabolite clustering heatmap further demonstrated that these modules were enriched in the SLEL-treated group, whereas the green and cyan modules were more abundant in the model group. These findings indicate that different metabolite modules play distinct roles in inflammation and anti-inflammatory processes, with the pink, red, and magenta modules likely harboring key metabolites mediating the therapeutic effects (Figure 7H,I).

### 2.9. SLEL Attenuates Inflammation via Dual Suppression of PI3K-Akt and MAPK-NF-κB Pathways

**Figure 8:**
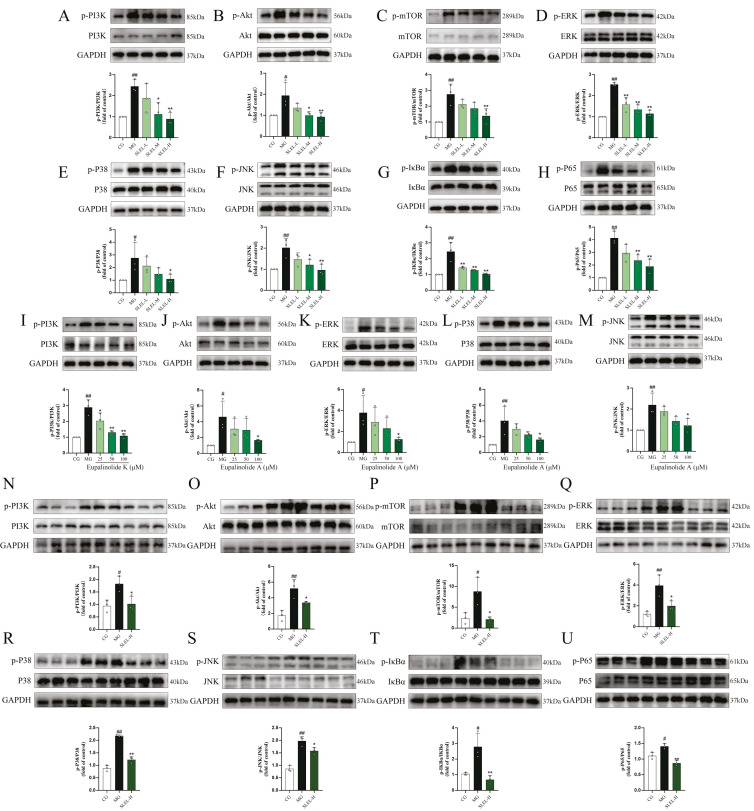
The anti-inflammatory activity of SLEL is associated with the modulation of key molecular targets in the PI3K-Akt and MAPK-NF-κB signaling pathways. SLEL effectively modulates key molecular targets in the PI3K-Akt and MAPK-NF-κB signaling pathways in A549 cells (n = 3), including (**A**) PI3K, (**B**) Akt, (**C**) mTOR, (**D**) ERK, (**E**) P38, (**F**) JNK, (**G**) IκBα, and (**H**) P65. Eupalinolide K significantly reduced (**I**) PI3K phosphorylation. Eupalinolide A markedly inhibited phosphorylation of (**J**) Akt, (**K**) ERK, (**L**) P38, and (**M**) JNK. SLEL effectively modulates key molecular targets in the PI3K-Akt and MAPK-NF-κB signaling pathways in ALI-rats (n = 3), including (**N**) PI3K, (**O**) Akt, (**P**) mTOR, (**Q**) ERK, (**R**) P38, (**S**) JNK, (**T**) IκBα, (**U**) P65. ^#^ *p* < 0.05, ^##^ *p* < 0.01 compared to the CG. * *p* < 0.05, ** *p* < 0.01 compared to the MG.

Mechanistic studies in LPS-stimulated A549 cells and an ALI rat model demonstrated that SLEL exerted a dose-dependent inhibitory effect on the PI3K-Akt and MAPK-NF-κB signaling pathways. In A549 cells, compared with the MG group, SLEL markedly suppressed LPS-induced phosphorylation of PI3K (*p* < 0.01), Akt (*p* < 0.01), mTOR (*p* < 0.01), ERK (*p* < 0.01), P38 (*p* < 0.05), JNK (*p* < 0.01), IκBα (*p* < 0.01), and P65 (*p* < 0.01) (Figure 8A–H). In line with the network pharmacology predictions, the regulatory effects of bioactive compounds Eupalinolide A and Eupalinolide K were further evaluated on key molecular targets within the PI3K-Akt and MAPK-NF-κB signaling pathways. Compared with the MG group, Eupalinolide K (100μM) significantly reduced PI3K (*p* < 0.01) phosphorylation, while Eupalinolide A (100μM) markedly decreased the phosphorylation of Akt (*p* < 0.05), ERK (*p* < 0.05), P38 (*p* < 0.05), and JNK (*p* < 0.05) (Figure 8I–M). In ALI rats, SLEL similarly reversed the LPS-induced upregulation of phosphorylation of PI3K (*p* < 0.05), Akt (*p* < 0.05), mTOR (*p* < 0.05), ERK (*p* < 0.05), P38 (*p* < 0.01), JNK (*p* < 0.05), IκBα (*p* < 0.01), and P65 (*p* < 0.01) (Figure 8N–U). These findings indicate that SLEL consistently suppresses pro-inflammatory signaling across multiple models, highlighting its therapeutic potential through dual-pathway synergistic regulation.

## 3. Discussion

The persistent clinical challenge of ALI and the limitations of current therapies have propelled the development of safe and efficacious natural medicines to the forefront of translational medical research. In this study, we identified the potential of SLEL as a multi-target therapeutic agent for ALI. SLEL effectively reduced the production of LPS-induced pro-inflammatory cytokines both in vitro and in vivo. In a rat model of LPS-induced ALI established Via intratracheal instillation, SLEL also alleviated pulmonary edema, alveolar hemorrhage, and inflammatory cell infiltration, with the high-dose group demonstrating superior efficacy compared to DEX. These observations indicate that SLEL exerts significant protective effects against LPS-induced acute lung injury.

In ALI, macrophages and neutrophils are activated, releasing large amounts of cytokines and reactive oxygen species, leading to severe inflammation and tissue injury. ALI also disrupts the alveolar epithelial barrier [15]. The severity of lung injury depends on the interactions between inflammatory cells and alveolar epithelial cells, as well as the extent of epithelial cell damage. Therefore, in this study, LPS was used to induce type II alveolar epithelial cells (A549) to mimic epithelial cell injury during ALI [16]. Our results demonstrated that SLEL significantly suppressed the mRNA expression of TNF-α, IL-6, and IL-1β—cytokines closely associated with patient mortality [17]—in A549 cells during inflammatory injury, thereby alleviating epithelial inflammation. These findings highlight the potential clinical value of SLEL in the treatment of ALI.

**Figure 9:**
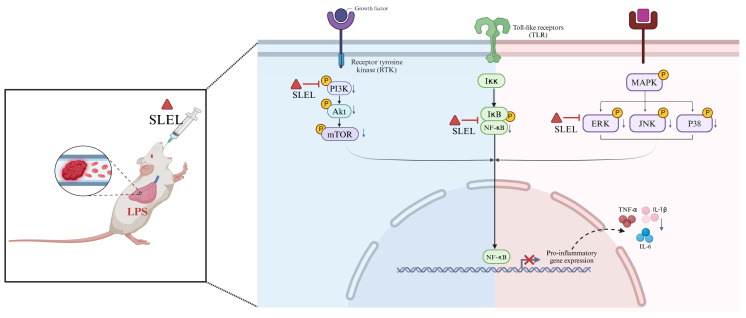
The protective effects of SLEL by modulating the PI3K-Akt and MAPK-NF-κB pathway against ALI.

The protective effects of SLEL against ALI are mediated by modulation of the PI3K-Akt and MAPK-NF-κB signaling pathways. This study integrated network pharmacology, transcriptomics, metabolomics, and 16S rRNA gene sequencing to establish a self-correcting framework combining computational predictions with empirical validation [18,19]. By linking molecular mechanisms to phenotypic outcomes, this strategy revealed that SLEL protects against ALI through simultaneous regulation of PI3K-Akt and MAPK-NF-κB pathways. The PI3K-Akt pathway plays a key role in maintaining macrophage homeostasis [20], the MAPK pathway contributes to the synthesis of inflammatory mediators and is a potential target for anti-inflammatory therapy [21], and NF-κB-mediated macrophage overactivation is closely associated with inflammatory tissue injury [22]. Consistently, our results showed that SLEL inhibited the LPS-induced phosphorylation of PI3K, Akt, mTOR, ERK, JNK, P38, IκBα, and P65 both in vitro and in vivo, confirming its suppressive effects on the PI3K-Akt and MAPK-NF-κB axes (Figure 9). In addition, network pharmacology analysis and molecular docking revealed that Eupalinolide A and Eupalinolide K exhibited strong binding affinity with key protein targets in the PI3K-Akt and MAPK signaling pathways. Based on these predictions, we further explored and experimentally validated their molecular roles in anti-inflammatory activity. The results demonstrated that both compounds significantly inhibited the phosphorylation of critical proteins within these pathways. These findings further support the notion that the protective effects of SLEL arise from the synergistic actions of multiple constituents, while certain key sesquiterpene lactones can serve as representative components linking chemical identity to pharmacological activity.

Notably, the traditional Chinese medicine theory of “the lung and large intestine being interior-exteriorly related” aligns closely with the modern concept of the gut–lung axis, a bidirectional communication system mediated by microbiota, metabolites, and immune networks [23]. During the development of ALI, pulmonary microbiota can translocate into the bloodstream, disrupting gut microbial homeostasis and exacerbating pulmonary inflammation Via distal mucosal immune dysregulation. SLEL counteracts this pathological process by upregulating anti-inflammatory metabolites while downregulating inflammation-associated metabolites, which in turn remodel the gut microbiota through metabolite–microbe interactions [24]. Specifically, SLEL enriched beneficial genera such as *norank_f__Muribaculaceae* and *Akkermansia*, while suppressing Bacteroides, which are linked to mucosal barrier dysfunction [25]. Further correlation analyses suggested that SLEL reshaped the microbiota–metabolite–cytokine network. Collectively, these multidimensional findings indicate that SLEL may restore gut–lung axis homeostasis by modulating gut microbiota and metabolites, thereby alleviating ALI-driven excessive pulmonary inflammation.

In summary, this study demonstrated that SLEL exerts significant protective effects against ALI. SLEL reduced pro-inflammatory cytokine release, alleviated pulmonary edema, hemorrhage, and inflammatory infiltration, and improved epithelial barrier function. Mechanistically, SLEL simultaneously suppressed the PI3K-Akt and MAPK-NF-κB pathways and restored gut microbiota composition and metabolite balance, thereby maintaining gut–lung axis homeostasis. Representative constituents Eupalinolide A and Eupalinolide K further confirmed direct inhibition of key signaling proteins, linking chemical identity to pharmacological activity.

Although this study revealed the multi-target mechanisms of SLEL in alleviating acute lung injury, several limitations should be noted. First, while we verified that Eupalinolide A and Eupalinolide K inhibit phosphorylation of key proteins in the PI3K-Akt/MAPK pathways, these findings only partially account for the overall pharmacological effects of SLEL. The potential roles of other bioactive constituents and their synergistic interactions remain to be elucidated for a more comprehensive understanding of SLEL’s effects. Second, our analyses were primarily cross-sectional, capturing static changes in gut microbiota and metabolites, while their temporal dynamics remain unclear. Future studies should incorporate time-series metagenomics to track the progression of microbial remodeling. In addition, the causal relationship between gut microbial alterations and pulmonary anti-inflammatory effects should be validated through antibiotic depletion and fecal microbiota transplantation experiments. Moreover, given that neutrophil activation and recruitment play a critical role in the pathogenesis of ALI, whether SLEL can modulate neutrophil responses remains to be further explored. Furthermore, the pathophysiology of ALI also involves complex processes such as apoptosis, autophagy, and necrosis, and it remains unclear whether SLEL exerts regulatory effects on these cell death pathways. Finally, single-cell spatial transcriptomics may help resolve the spatial heterogeneity of signaling pathways at the sub-organ level, thereby deepening our mechanistic understanding of SLEL.

## 4. Materials and Methods

Materials and reagents

*Eupatorium lindleyanum* DC. was purchased from Xuyi Defeng Traditional Chinese Medicine Planting Co., Ltd. (Huai’an, China) and authenticated by Professor Xianyuan He at the College of Traditional Chinese Medicine of Chongqing Medical University. Taxonomic details and the accepted name were confirmed as ID: wfo-0000021725 in the World Flora Online: http://www.worldfloraonline.org/ (accessed on 6 May 2025). The EL medicinal materials were refluxed twice with 70% ethanol under reduced pressure and heated, and the concentrated extract was vacuum freeze-dried as the total EL powder extract. Then, the EL powder was fully dissolved with pure water, and was sequenced by polyamide resin and D101 macroporous adsorption resin. After adsorption, the components adsorbed by D101 macroporous adsorption resin were eluted with anhydrous ethanol. The eluted liquid was vacuum freeze-dried to obtain SLEL powder, which underwent compositional analysis using ultra-high-performance liquid chromatography coupled with time-of-flight mass spectrometry (UPLC-QTOF-MS).

Lipopolysaccharide (LPS, Escherichia coli 055:B5) and dexamethasone acetate tablets (Batch No. 200923) were procured from Sigma-Aldrich (St. Louis, MO, USA) and Zhejiang Xianju Pharmaceutical Co., Ltd. (Hangzhou, China), respectively. Molecular biology reagents, including SYBR Green quantitative real-time polymerase chain reaction (qRT-PCR) Master Mix (Cat# HY-K0522) and RT Master Mix for qPCR (Cat# HY-K0510), were sourced from Med Chem Express (Monmouth Junction, NJ, USA). ELISA kits for TNF-α (EHJ-20039r), IL-6 (EHJ-10464h, EHJ-20746r), and IL-1β (EHJ-20537r). Antibodies against Akt (4691), p-JNK (4668), P38 (9212), p-P38 (4511), IκBα (4812) and p-IκBα (2859) were sourced from Cell Signaling Technology (Boston, USA), whereas mTOR (T55306), p-mTOR (T55996), ERK (T40071), P65 (TA5006), p-P65 (TP70621) was purchased from Abmart (Shanghai, China). Furthermore, JNK (YT2440), p-ERK (YP0101), p-Akt (YP0006), and GAPDH (YN5585) were provided by ImmunoWay (California, USA).

UPLC-MS/MSn analysis

Chromatographic separation was achieved using a UPLC system with binary mobile phases: (A) acetonitrile and (B) 0.1% (*v*/*v*) aqueous formic acid. The gradient elution protocol comprised four phases: (1) 0–25 min: Isocratic at 10% A and 90% B, (2) 25–27 min: Linear gradient from 10% to 70% A and 90% to 30% B, (3) 27–31 min: Step gradient to 90% A and 10% B, (4) 31–36 min: Re-equilibration to initial conditions (10% A and 90% B). Operating parameters were maintained at a flow rate of 0.25 mL·min^−1^, column temperature of 35 °C, and injection volume of 0.1 µL.

Network pharmacology analysis of SLEL bioactive compounds

Computational Target Prediction and Analysis. Two-dimensional structures of compounds (from UPLC-QTOF-MS) were retrieved from PubChem (https://pubchem.ncbi.nlm.nih.gov/, accessed on 6 May 2025). Targets were predicted using SEA Search Server (https://sea16.docking.org/, accessed on 6 May 2025) and SwissTargetPrediction (swisstargetprediction.ch), retaining predictions with probability > 0. Identified targets were standardized Via UniProt (https://www.uniprot.org/, accessed on 6 May 2025) for unified nomenclature.

Disease Target Collection and Intersection Analysis. ALI-related targets were compiled from GeneCards (https://www.genecards.org/, accessed on 6 May 2025), DrugBank (https://go.drugbank.com/, accessed on 6 May 2025), TTD (https://db.idrblab.net/ttd/, accessed on 6 May 2025), OMIM (https://www.omim.org/, accessed on 6 May 2025), and PharmGKB (https://www.pharmgkb.org/, accessed on 6 May 2025), then merged, deduplicated, and standardized using UniProt. Overlapping genes between SLEL and ALI were visualized via a Venn diagram as potential therapeutic targets.

Protein–protein interaction (PPI) Network and Functional Enrichment. Intersection targets were analyzed using STRING (confidence score > 0.9, https://cn.string-db.org/, accessed on 6 May 2025) to build a PPI network, refined in Cytoscape 3.7.2. Hub genes were identified Via Cytohubba topological analysis. Metascape enrichment and Kyoto Encyclopedia of Genes and Genomes (KEGG) pathway analysis (top 30 pathways) elucidated biological mechanisms.

Cell culture

A549 cells (Wuhan Pricella Biotechnology Co., Ltd. Wuhan, China) were cultured in Ham’s F-12K medium supplemented with 10% heat-inactivated fetal bovine serum (FBS) and 1% penicillin-streptomycin, and maintained at 37 °C in a humidified atmosphere containing 5% CO_2_. Cells were subcultured up to passage 5 before use in experiments [26]. For cytotoxicity analysis, A549 cells were exposed to graded concentrations of SLEL for 24 h. Cell viability was assessed using the CCK-8 assay: 10 μL of CCK-8 solution was added to each well, followed by incubation for 1 h at 37 °C under 5% CO_2_. Absorbance at 450 nm was then measured with a microplate reader, and results were normalized to the untreated control group (set as 100% viability).

Animal treatment

Male Sprague-Dawley rats (6–8 weeks, 180–220 g) from Chongqing Medical University’s Animal Center (License No. SYXK [Yu] 2022–0010) were acclimatized for 1 week and randomized into six groups (n = 6): control (CG), LPS-induced ALI model (MG), dexamethasone (DEX, 5 mg/kg), and low/medium/high-dose SLEL (50/100/200 mg/kg). Treatments were administered orally for 7 days. ALI was induced on day 7 Via intratracheal LPS instillation (5 mg LPS dissolved in 1 mL normal saline, and the volume of injection is 1 mL/kg) as described [14]. After 24 h, bronchoalveolar lavage fluid (BALF), serum, and lung tissues were collected. Serum and lung samples were stored at −80 °C; partial lung tissues were fixed in 10% neutral-buffered formalin for histopathology. All procedures adhered to institutional guidelines (Ethics Approval: IACUC-CQMU-2025-0029, approved on 15 January 2025).

Pharmacodynamic evaluation of SLEL

Body weights were recorded 24 h post-LPS exposure. Post-lavage, lungs were weighed to calculate the lung coefficient (lung-to-body weight ratio × 100%). The left upper lobe’s wet weight (W) was measured, dried at 60 °C for 48 h to determine dry weight (D), and the W/D ratio was calculated. The left inferior lobe was fixed in 4% paraformaldehyde, processed through graded ethanol and xylene, and paraffin-embedded for 5 µm sectioning. The tissue sections were then stained with hematoxylin and eosin (H&E) and visualized under a light microscope. The entire surface of the lung was analyzed for inflammation and damage, and was scored as follows: normal lung (0), hemorrhage (on a 0–1 scale), peribronchial infiltration (on a 0–1 scale), interstitial edema (on a 0–2 scale), pneumocyte hyperplasia (on a 0–3 scale) and intra- alveolar infiltration (on a 0–3 scale) [27].

Enzyme-linked immunosorbent assay (ELISA)

Serum, BALF, and cell supernatants (A549 cells) were collected post-treatment and assayed for IL-6, IL-1β, and TNF-α levels using commercial ELISA kits (Xiamen Huijia Biotechnology Co., Ltd., Xiamen, China) per manufacturer’s protocols.

Western blot analysis

In line with the previous results, a Western blot was performed [28]. Briefly, proteins from cells/lung tissues were extracted using RIPA buffer with protease/phosphatase inhibitors (Beyotime Biotechnology Co., Ltd., Shanghai, China), quantified Via BCA assay, and separated on SDS-PAGE gels. After transfer to polyvinylidene fluoride (PVDF, Millipore, Boston, MA, USA) membranes, blocking with 5% non-fat milk for 1h, and incubation with primary antibodies (4 °C, overnight) and HRP-conjugated secondary antibodies, signals were detected using ECL reagents (Advansta, CA, USA). Antibodies included Akt (1:1000), p-JNK (1:1000), P38 (1:1000), p-P38 (1:1000), IκBα (1:1000), p-IκBα (1:1000), mTOR (1:1000), p-mTOR (1:1000), ERK (1:1000), P65 (1:1000), p-P65 (1:1000), JNK (1:1000), ERK (1:1500), p-Akt (1:1000), and GAPDH (1:5000). Quantification used ImageJ (https://imagej.net/ij/download.html, accessed on 6 June 2025).

qRT-PCR

Total RNA from lung tissues, A549 cells was extracted with TRIzol reagent, reverse-transcribed into cDNA (RT Master), and analyzed Via SYBR Green qPCR. Primer sequences are listed in Supplementary Table S1. Gene expression was normalized to GAPDH using the 2^−ΔΔCT^ method.

Data and Statistical Analysis

Data (mean ± SD, ±s) were analyzed using GraphPad Prism 9. Differences between the two groups were assessed by an unpaired t-test, while multi-group comparisons were made using one-way ANOVA. Statistical significance was defined as a p-value less than 0.05, with significance levels denoted as ^#^ *p* < 0.05 and ^##^ *p* < 0.01 compared to the CG, * *p* < 0.05 and ** *p* < 0.01 compared to the MG.

## 5. Conclusions

This study systematically elucidated the protective effects and molecular mechanisms of SLEL in alleviating acute lung injury. The results demonstrated that SLEL significantly reduced the release of pro-inflammatory cytokines, including TNF-α, IL-6, and IL-1β, thereby mitigating the inflammatory injury of alveolar epithelial cells and overall lung tissue damage. Mechanistically, SLEL exerts dual suppression of the PI3K-Akt and MAPK-NF-κB pathways, accompanied by the restoration of gut metabolites and microbiota composition, which helps maintain gut–lung axis homeostasis and further attenuate ALI. Notably, representative constituents Eupalinolide A and Eupalinolide K were confirmed to directly inhibit phosphorylation of key signaling proteins, suggesting their important contribution to the overall efficacy of SLEL. In conclusion, SLEL, as a multi-component and multi-target natural agent, shows promising potential for the prevention and treatment of acute lung injury.

## Data Availability

The data will be available from the corresponding author. The data are not publicly available due to ongoing related studies and institutional restrictions but are available from the corresponding author upon reasonable request.

## Supplementary Materials

The following supporting information can be downloaded at: https://www.mdpi.com/article/10.3390/ph18101523/s1, Figure S1: Total ion current chromatograms showing components of total sesquiterpene lactones of *Eupatorium lindleyanum* DC. with the positive (red) and negative (purple) ion modes.; Table S1: Sequences of primers used for quantitative real-time PCR (qRT-PCR) analysis; Table S2: The main compounds identified in SLEL by UPLC-Q/TOF-MSn.

## Abbreviations

The following abbreviations are used in this manuscript:

ALI	Acute lung injury
ARDS	Acute respiratory distress syndrome
BALF	Bronchoalveolar lavage fluid
BCA	Bicinchoninic acid assay
CCK-8	Cell counting kit-8
CO_2_	Carbon dioxide
DMEM	Dulbecco’s modified Eagle medium
ECL	Enhanced chemiluminescence
ELISA	Enzyme-linked immunosorbent assay
ERK	Extracellular regulated protein kinases
Go	Gene ontology
GSEA	Gene set enrichment analysis
IκBα	Inhibitor of nuclear factor kappa B alpha
IL-1β	Interleukin-1β
IL-6	Interleukin-6
JNK	c-Jun N-terminal kinase
KEGG	Kyoto encyclopedia of genes and genomes
LPS	Lipopolysaccharide
MAPK	Mitogen-activated protein kinase
mTOR	Mechanistic target of rapamycin
NF-κB	Nuclear factor kappa-B
PCA	Principal component analysis
PI3K	Phosphatidylinositol 3-kinase
PMA	Phorbol myristate acetate
PPI	Protein–protein interaction
ROC	Receiver operating characteristic
RPMI	Roswell Park Memorial Institute
SLEL	Sesquiterpenoid lactones of *Eupatorium lindleyanum* DC.
TCM	Traditional Chinese medicine
TNF-α	Tumor necrosis factor-α
UPLC-QTOF-MS	Ultra-high-performance liquid chromatography coupled with time-of-flight mass spectrometry
VIP	Variable importance in projection
WGCNA	Weighted gene co-expression network analysis
