# Supplementary material for: Multi-Omics and Experimental Insights into the Protective Effects of Sesquiterpenoid Lactones from Eupatorium lindleyanum DC. in Acute Lung Injury: Regulation of PI3K-Akt and MAPK-NF-κB Pathways
Source: Pharmaceuticals (Basel). 2025 Oct 10;18(10):1523. doi: 10.3390/ph18101523 (PMC12567390; doi:10.3390/ph18101523)
Supplement: Supplementary file 1 [file pharmaceuticals-18-01523-s001.zip › pharmaceuticals-3902143-supplementary/Supplementary Table S1.pdf]

Gene	Forward primer	Reverse primer
GAPDH (Rat)	ACAGCAACAGGGTGGTGGAC	TTTGAGGGTGCAGCGAACTT
TNF-α (Rat)	CACCACGCTCTTCTGTCTACTGAAC	TGGGCTACGGGCTTGTCACTC
IL-6 (Rat)	ACTTCCAGCCAGTTGCCTTCTTG	TGGTCTGTTGTGGGTGGTATCCTC
IL-1β (Rat)	AATCTCACAGCAGCATCTCGACAAG	TCCACGGGCAAGACATAGGTAGC
GAPDH (Human)	GGAGCGAGATCCCTCCAAAAT	GGCTGTTGTCATACTTCTCATGG
TNF-α (Human)	AGCCCTGGTATGAGCCCATCTATC	TCCCAAAGTAGACCTGCCCAGAC
IL-6 (Human)	GGTGTTGCCTGCTGCCTTCC	GTTCTGAAGAGGTGAGTGGCTGTC
IL-1β (Human)	GCCAGTGAAATGATGGCTTATT	AGGAGCACTTCATCTGTTTAGG
