# Supplementary material for: Multi-Omics and Experimental Insights into the Protective Effects of Sesquiterpenoid Lactones from Eupatorium lindleyanum DC. in Acute Lung Injury: Regulation of PI3K-Akt and MAPK-NF-κB Pathways
Source: Pharmaceuticals (Basel). 2025 Oct 10;18(10):1523. doi: 10.3390/ph18101523 (PMC12567390; doi:10.3390/ph18101523)
Supplement: Supplementary file 1 [file pharmaceuticals-18-01523-s001.zip › pharmaceuticals-3902143-supplementary/Supplementary Table S2.pdf]

Supplementary Table 2. The main compounds identified in SLEL by UPLC-Q/TOF-MSn.

| NO | T(min) | Compound name | Formula | Calculated (Da) | Selected ion | Error (ppm) | Selected ion | Error (ppm) |
|---|---|---|---|---|---|---|---|---|
| A | 4.096 | [(8R)-4-oxido-2,3,5,6,7,8-hexahydro-1H-pyrrolizin-4-ium-1-yl]methyl 2-hydroxy-2-(1-hydroxyethyl)-3-methylbutanoate | $C_{15}H_{27}NO_5$ | 301.1889 | $[M+H]^+$ | 2.31 | $[M-H]^-$ | -0.72 |
| B | 4.836 | Dihydroconiferin | $C_{16}H_{24}O_8$ | 344.1471 | $[M+Na]^+$ | 1.62 | $[M-H]^-$ | -0.48 |
| C | 5.171 | (4R)-4-hydroxy-3,5,5-trimethyl-4-[(E)-3-[(2S,3S,4R,5R,6S)-3,4,5-trihydroxy-6-(hydroxymethyl)oxan-2-yl]oxybut-1-enyl]cyclohex-2-en-1-one | $C_{19}H_{30}O_8$ | 386.1941 | $[M+H]^+$ | -0.13 | $[M+HCOO]^-$ | -0.64 |
| D | 5.965 | 3-O-Feruloylquinic acid | $C_{17}H_{20}O_9$ | 368.1107 | $[M+H]^+$ | 2.88 | $[M-H]^-$ | -1.27 |
| E | 7.621 | (2R,3R,4S,5S,6R)-2-[[(2S,3R,4S)-2,4-bis(4-hydroxy-3,5-dimethoxyphenyl)oxolan-3-yl]methoxy]-6-(hydroxymethyl)oxane-3,4,5-triol | $C_{27}H_{36}O_{13}$ | 568.2156 | $[M+Na]^+$ | -0.23 | $[M+HCOO]^-$ | 0.15 |
| F | 8.743 | Blumenol C glucoside | $C_{19}H_{32}O_7$ | 372.2148 | $[M+H]^+$ | -0.15 | $[M-H]^-$ | -0.15 |
| G | 10.507 | Eupalinolide C | $C_{22}H_{28}O_8$ | 420.1784 | $[M+Na]^+$ | 0.74 | $[M+HCOO]^-$ | 1.38 |
| H | 11.165 | Eupalinolide K | $C_{20}H_{26}O_6$ | 362.1729 | $[M+H]^+$ | 1.51 | $[M+HCOO]^-$ | -2.21 |
| I | 11.570 | Eupalinolide O | $C_{22}H_{26}O_8$ | 418.1628 | $[M+H]^+$ | 0.97 | $[M+HCOO]^-$ | -1.46 |
| J | 11.783 | (1S,4aR)-5'-(2-hydroxyethyl)-1,5'-bis(hydroxymethyl)-1,4a,6-trimethylspiro[3,4,6,7,8,8a-hexahydro-2H-naphthalene-5,2'-oxolane]-2-ol | $C_{20}H_{36}O_5$ | 356.2563 | $[M+H]^+$ | 1.29 | $[M-H]^-$ | -1.71 |
| K | 12.386 | Eupalinolide H | $C_{22}H_{28}O_8$ | 420.1784 | $[M+H]^+$ | 0.52 | $[M-H]^-$ | -2.03 |
| L | 12.777 | Eupalinilide B | $C_{20}H_{24}O_6$ | 360.1573 | $[M+Na]^+$ | 0.50 | $[M+HCOO]^-$ | 1.74 |
| M | 13.906 | (5E,9E,13Z)-3-(1,2-dihydroxy-2-methylpropyl)-15-hydroxy-13-(hydroxymethyl)-5,9-dimethylpentadeca-5,9,13-trienoic acid | $C_{22}H_{38}O_6$ | 398.2668 | $[M+H]^+$ | 2.82 | $[M-H]^-$ | -0.29 |
| N | 14.406 | Eupalinolide P | $C_{22}H_{26}O_8$ | 418.1628 | $[M+H]^+$ | 2.35 | $[M-H]^-$ | -2.61 |
| O | 14.621 | Eupalinolide A | $C_{24}H_{30}O_9$ | 462.1890 | $[M+H]^+$ | 2.15 | $[M-H]^-$ | -2.17 |
| P | 15.081 | (6E,10E,14Z)-4,16-dihydroxy-14-(hydroxymethyl)-3-(2-hydroxypropan-2-yl)-6,10-dimethylhexadeca-6,10,14-trienoic acid | $C_{22}H_{38}O_6$ | 398.2668 | $[M+Na]^+$ | 2.01 | $[M+HCOO]^-$ | -2.72 |

| | | | | | | | | |
|---|---|---|---|---|---|---|---|---|
| Q | 15.935 | Eupalinolide B | $C_{24}H_{30}O_9$ | 462.1890 | $[M+H]^+$ | 1.34 | $[M-H]^-$ | -3.79 |
| R | 16.436 | Eupalinolide J | $C_{22}H_{28}O_7$ | 404.1835 | $[M+H]^+$ | -0.97 | $[M-H]^-$ | -2.75 |
